# Supplementary material for: Staphylococcal Enterotoxin C—An Update on SEC Variants, Their Structure and Properties, and Their Role in Foodborne Intoxications
Source: Toxins (Basel). 2020 Sep 10;12(9):584. doi: 10.3390/toxins12090584 (PMC7551944; doi:10.3390/toxins12090584)
Supplement: Supplementary file 1 [file toxins-12-00584-s001.zip › toxins-905136 - supplementary.docx]

Supplementary Materials: Staphylococcal Enterotoxin C—an Update on SEC Variants, Structure and Properties, and Their Role in Foodborne Intoxications

Danai Etter, Jenny Schelin, Markus Schuppler and Sophia Johler


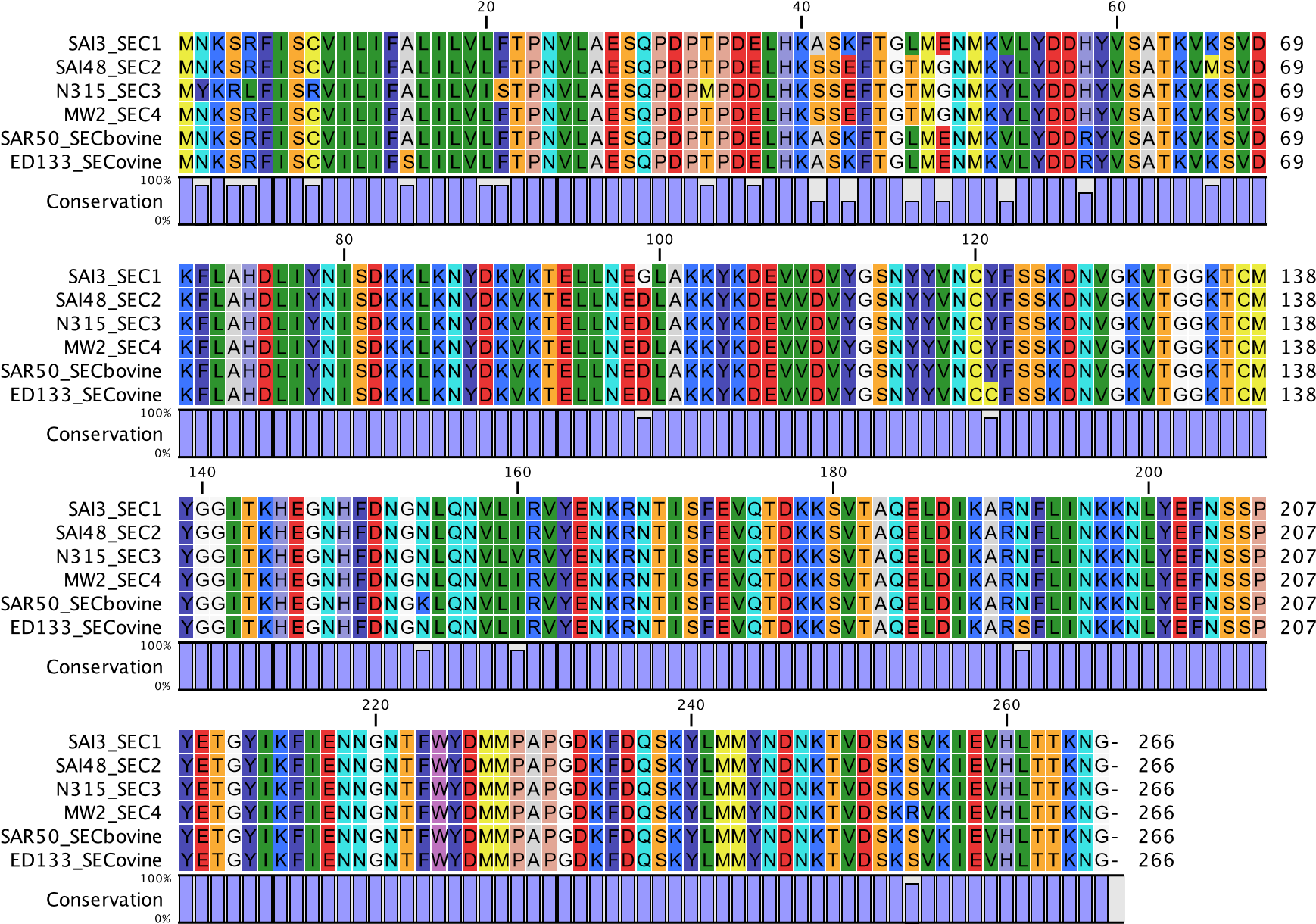


**Figure S1.** SEC protein sequence alignments.
